# Supplementary material for: ProteinShader: illustrative rendering of macromolecules
Source: BMC Struct Biol. 2009 Mar 30;9:19. doi: 10.1186/1472-6807-9-19 (PMC2672931; doi:10.1186/1472-6807-9-19)
Supplement: Additional file 1 — ProteinShader program without source code. This compressed file contains the complete ProteinShader program including associated libraries, but no source code. A README.txt file gives an overview of the ProteinShader distribution, and the index.html file in the help subdirectory has directions on getting started with the program as well as a set of tutorials. [file 1472-6807-9-19-S1.zip › ProteinShader-beta-0_9_4-binary/help/api/org/proteinshader/structure/visitor/BoundsVisitor.html]

BoundsVisitor (ProteinShader API)


|  |  |  |
| --- | --- | --- |
| **Overview** \| **Package** \| **Class** \| **Use** \| **Tree** \| **Deprecated** \| **Index** \| **Help** | |  |
| **PREV CLASS**   **NEXT CLASS** | **FRAMES**    **NO FRAMES**     **All Classes** |  |
| SUMMARY: NESTED \| FIELD \| CONSTR \| METHOD | DETAIL: FIELD \| CONSTR \| METHOD |  |


---


## org.proteinshader.structure.visitor Class BoundsVisitor

```
java.lang.Object
  org.proteinshader.structure.visitor.Visitor
      org.proteinshader.structure.visitor.BoundsVisitor
```

---

``` public class BoundsVisitor extends Visitor ```

Knows how to find the min and max xyz-coordinate values for all Atoms
in each Model.

---

**Constructor Summary**

`BoundsVisitor()`
  
          Constructs a BoundsVisitor.


| **Method Summary** | |
| --- | --- |
| `void` | `visit(Atom atom)`             Checks xyz-coordinates to look for any new min or new max. |
| `void` | `visit(Model model)`             Finds the min and max values for x, y, and z of the Model and then calls setter methods on the Model so that these values are remembered. |

**Methods inherited from class org.proteinshader.structure.visitor.Visitor**

`clear, includeAAHetAndWater, setDebug, setMode, setMode, setMode, setMode, setMode, setMode, setVisitSegments, visit, visit, visit, visit, visit, visit, visit, visit, visit, visit`

**Methods inherited from class java.lang.Object**

`clone, equals, finalize, getClass, hashCode, notify, notifyAll, toString, wait, wait, wait`

**Constructor Detail**

### BoundsVisitor

```
public BoundsVisitor()
```

:   Constructs a BoundsVisitor.


**Method Detail**

### visit

```
public void visit(Model model)
           throws VisitorException
```

:   Finds the min and max values for x, y, and z of the Model and then
    calls setter methods on the Model so that these values are
    remembered.

    :   **Overrides:**: `visit` in class `Visitor`
    :   **Parameters:**: `model` - the Model to test. **Throws:**: `VisitorException` - if an error occurs during the traversal.

---


### visit

```
public void visit(Atom atom)
           throws VisitorException
```

:   Checks xyz-coordinates to look for any new min or new max.

    :   **Overrides:**: `visit` in class `Visitor`
    :   **Parameters:**: `atom` - the Atom to test. **Throws:**: `VisitorException` - if an error occurs during the traversal.


---


|  |  |  |
| --- | --- | --- |
| **Overview** \| **Package** \| **Class** \| **Use** \| **Tree** \| **Deprecated** \| **Index** \| **Help** | |  |
| **PREV CLASS**   **NEXT CLASS** | **FRAMES**    **NO FRAMES**     **All Classes** |  |
| SUMMARY: NESTED \| FIELD \| CONSTR \| METHOD | DETAIL: FIELD \| CONSTR \| METHOD |  |


---

# *Copyright © 2007-2008*
